# Education Research: The MANET Project: Museum Art in Neurology Education Training

**DOI:** 10.1212/NE9.0000000000200170

**Published:** 2024-11-13

**Authors:** Tatiana Greige, David Odo, Camran Mani, Stephanie Bissonnette, Pria Anand

**Affiliations:** From the Department of Neurology (T.G., P.A.), Boston Medical Center, MA; Georgia Museum of Art (D.O.), Athens; Harvard Art Museums (C.M.), Cambridge, MA; and Department of Neurology (S.B.), Virginia Commonwealth University, Richmond.

## Abstract

**Background and Objectives:**

Multiple studies have shown that visual arts training has improved observational and communication skills and empathy among medical students and resident physicians. The benefits of such training for neurology residents remain scarce. This project aims to introduce neurology residents to the world of visual arts, improve their observational skills, foster their empathic skills, and provide them with a unique space for self-expression.

**Methods:**

Neurology residents at an urban tertiary academic medical center in the northeastern United States received multiple custom-designed art observation training sessions at the Fogg Museum. Sessions were led by professional art educators and involved visiting multiple galleries in-person. Residents completed preintervention and postintervention tests to assess for change in their observational skills. The test was composed of artwork, MRIs, and videos with neurologic findings, which were all graded using a priori rubrics. The primary outcome was the difference between preintervention and postintervention total test scores. Secondary outcomes included the differences between preintervention and postintervention scores for art imagery and clinical imagery. Two-tailed paired Student t test was used.

**Results:**

Seventeen neurology residents attended the museum art sessions throughout the academic year and 12 (71%) residents completed both the preintervention and postintervention tests. Observational skills, as calculated by the total score, improved significantly between the preintervention and postintervention tests (mean score 22.75 vs 33.5, respectively, *p* = 0.00005). Most residents noted a subjective improvement in their communication and observational skills and an increase in their empathy skills. All residents noted feeling more comfortable with the notion of ambiguity in a clinical setting.

**Discussion:**

Residents' observational skills improved significantly throughout the academic year. This study emphasizes the importance of visual arts in neurology training. Art can further develop residents' observational skills, foster their empathy and humanity, and provide them with a safe space for self-reflection and personal growth.

## Introduction

Art and neurology have long been intertwined. Renaissance artwork by Leonardo da Vinci,^1^ Vesalius,^2^ Michelangelo,^3^ and others depicted the nuances of human anatomy and pathology with remarkable accuracy, whereas impressionism, cubism, and other artistic movements exploit the unique features of human vision and perception.^4^ Just as artists rely on an intuitive understanding of neuroscience, neuroscientists, such as Santiago Ramón y Cajal, have long documented their findings with artistic renderings of the brain, giving rise to Cajal's seminal “neuron doctrine” and other key scientific observations.^5^

In turn, the art of neurologic diagnosis requires meticulous observation, with careful attention paid to a patient's history, physical examination findings, and neuroimaging.^6^ However, resident physicians face heavy workloads, burnout, and emotional exhaustion because of the challenging nature of their training.^7^ These stressors have the potential to hinder residents' observational skills. Moreover, maintaining empathy and a patient-centered approach to care while navigating rigorous schedules and a complex health care system may be challenging and emotionally draining.^8^

Multiple studies have shown that visual arts training improves observational skills, communication, and empathy among medical students^9-12^ and ophthalmology,^13^ nuclear medicine, radiology,^14^ and dermatology^15^ residents. However, studies examining the benefits of visual arts training for neurology residents remain scarce.^16^

The Museum Art in Neurology Education Training (MANET) project aims to introduce neurology residents to the world of visual arts, improve their observational skills, foster their empathic skills, develop their tolerance for ambiguity, and provide them with a unique space for self-expression and personal growth through museum-based sessions.

## Methods

### Standard Protocol Approvals, Registrations, and Patient Consents

This study was exempt from Institutional Review Board approval (IRB number H-43116). Written consent was obtained for study participation. Participation was voluntary, and responses were anonymous.

### Setting and Study Participants

MANET sessions were held at Harvard University's Fogg Museum, an academic art institution with extensive collections and educational programming focused on “critical looking and thinking.” Study participants were resident physicians undergoing training in both adult and pediatric neurology in the Boston University Neurology Residency Program. Residents rotate at both the Boston Veteran's Affairs Medical Center and the Boston Medical Center, an academic, tertiary care, safety-net medical center. There are 7 adult neurology residents per class (postgraduate year 2 through 4) and 2 pediatric neurology residents for a total of 23 neurology residents per academic year. Residents have protected education time every Tuesday afternoon from 12 pm to 4 pm. When residents are at didactics, nurse practitioners and attendings cover the inpatient pagers. Residents are still responsible for clinical duties including writing clinical notes before and after didactics and are expected to return to their respective services after didactics until sign-out. Residents are excused from didactics if they are on night float rotations or are on vacation.

### Curriculum Development

The curriculum was developed in collaboration with the professional art educators at Harvard University's Fogg Museum and was guided by a framework of reflective practice. The program was offered without any associated costs. The first step included the choice of a broad topic (e.g., power, ambiguity or improvisation) that offered ample common ground and resonance between the history of art and present-day clinical practice. Prompts were then created to encourage participants to look closely at relevant works of art in the museum. Residents were also asked to reflect not only on how the specific topic chosen manifests itself in their personal and professional life but also on how the artworks' perspectives compared with their own. To deepen residents' exploration of the arts, hands-on activities were also created. The more informal and less hierarchal atmosphere of the museum was also leveraged to facilitate more reflective and philosophical conversations than are generally possible at the hospital.

### Art Sessions

**Figure 1:**
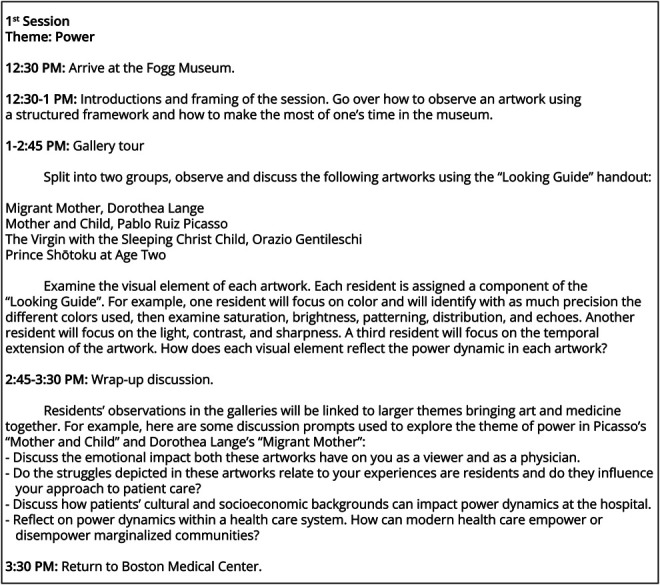
Structure of the First Session at the Fogg Museum

MANET sessions were held during the dedicated protected residency didactics block on Tuesday afternoons. Residents received a total of 3 custom-designed art observation training sessions between July 1, 2022, and June 30, 2023. Each session was 4 hours long and was led by at least 1 professional art educator. Sessions included in-person museum gallery visits and 1 hands-on session. The sessions touched on a range of themes, including power, ambiguity, and improvisation. An example of a session structure is illustrated in Figure 1.

### Preintervention and Postintervention Tests

**Figure 2:**
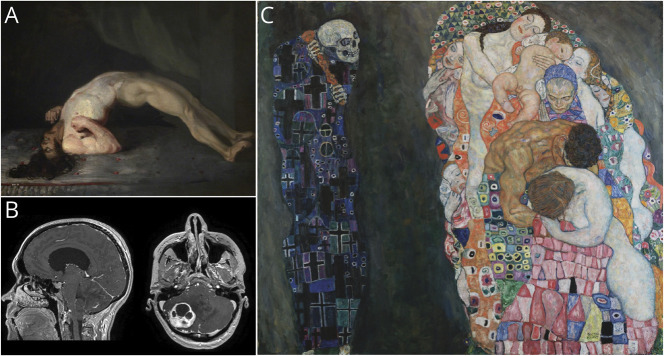
Examples of Clinical and Art Imagery Used in the Preintervention and Postintervention Tests (A) Charles Bell, The Wounded following the Battle of Corunna: Tetanus Following Gunshot Wounds, Photo © The Royal College of Surgeons of Edinburgh. Reprinted with permission from the Royal College of Surgeons of Edinburgh. (B) Sagittal and axial T1-weighted, postgadolinium images from a brain MRI demonstrating a hemangioblastoma of the right cerebellar hemisphere with associated mass effect and tonsillar herniation. (C) Gustav Klimt, Death and life, Photo © Leopold Museum, Vienna. Reprinted with permission from the Leopold Museum.

Residents completed preintervention and postintervention tests to assess for change in their observational skills. The tests included a combination of MRIs, clinical videos, and artworks (Figure 2). The preintervention test included 2 clinical vignettes with MRIs, 2 videos of clinical neurologic findings, and 2 artworks. The postintervention test included 3 clinical vignettes with MRIs and 2 artworks. The clinical and art imagery in the preintervention test differed from those in the postintervention test. Residents were asked to describe each artwork, MRI, or video, in the form of free text with no word limit. Artworks were chosen from a database of artworks available in the public domain and not exhibited at the Fogg Museum. MRIs were chosen from a database of de-identified MRIs. Videos were chosen from a personal database of videos of patients who consented to have their images used for resident teaching. Clinical imageries were chosen to ensure a diverse selection of pathologies covering different subspecialties within neurology including vascular neurology, neuro-oncology, neuro-immunology, neuro-infectious disease, and movement disorders. Participation was voluntary, and responses were anonymous.

The postintervention test also included a section for feedback and survey on any effects of the project on their observational and communication skills, empathy, or experience of ambiguity in medicine.

Only residents who completed both the preintervention and postintervention tests were included in this study. Tests were graded using a priori grading rubrics (eTables 1 and 2) by TG. The grader was masked to participant but not to time (preintervention or postintervention), and scores were reviewed by an independent third party. Although the third party did not participate in grading, they ensured that grading was fair and consistent with the a priori grading rubric. Each test had a maximum score of 50 points (20 points for art imagery and 30 points for clinical imagery). A personal and anonymous identifier, chosen by each resident, was used to link both tests to maintain anonymity during grading. When selecting the features to be included in the grading rubrics, the following components were prioritized: (1) the ability to describe the visual elements (color, shape, composition, symmetry) in an accurate and detailed way and (2) the ability to offer either an insightful interpretation of the artwork or to provide the correct diagnosis if a medical condition is portrayed. Points were awarded for specific observations that were predetermined. Each feature was assigned a certain number of points based on its perceived importance to the art or clinical imagery. A maximum of 2 additional points was awarded on a discretionary basis determined by the quality of the description, presence of personal interpretation, and identification of an observation that was not predetermined or included. The assessment tool was developed specifically for this project and has not undergone formal validation yet.

### Statistical Analysis

The primary outcome was the difference between preintervention and postintervention total test scores. Secondary outcomes included the differences between preintervention and postintervention scores for art imagery and clinical imagery. Two-tailed paired Student *t* test was used. *p* < 0.05 was considered statistically significant. Analysis was done using R. Figures were created using BioRender.com and Tableau.

### Data Availability

Anonymized data not published within this article may be shared at the request of any qualified investigator.

## Results

**Figure 3:**
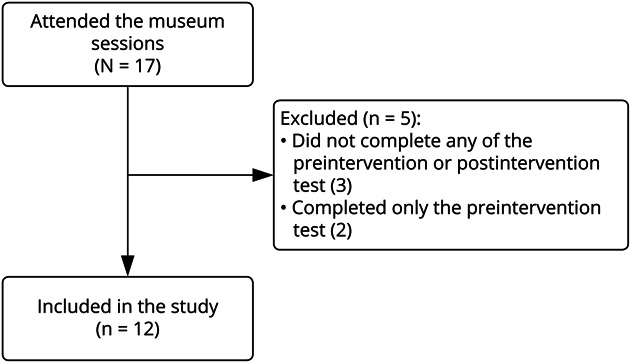
Study Flowchart

Seventeen of 23 neurology residents attended the museum art sessions throughout the academic year and 12 (71%) residents completed both the preintervention and postintervention tests. Five residents were excluded from this study (Figure 3). Most residents attended 2 or 3 sessions during the academic year. Characteristics of the residents included in this study are indicated in Table 1. The time period between completion of the preintervention and postintervention tests was 8 months.

**Table 1:** Baseline Characteristics of Neurology Residents Included in the Study

	Total (n = 12)
Sex	
Male	3 (25)
Female	9 (75)
Year in residency training	
PGY-2	5 (42)
PGY-3	7 (58)
Neurology residency type	
Adult neurology	11 (92)
Pediatric neurology	1 (8)
No. of sessions attended	
1	5 (42)
2	5 (42)
3	2 (16)

Abbreviation: PGY = postgraduate year.

Data are counts (n) and percentages (%).

**Figure 4:**
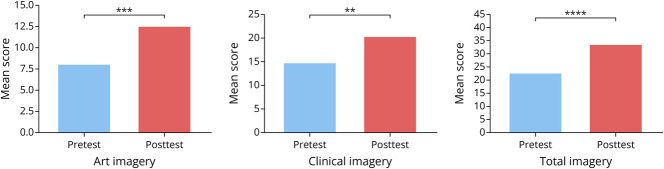
Mean Observation Scores on Preintervention and Postintervention Tests **p ≤ 0.01, ***p ≤ 0.001, ****p ≤ 0.0001.

Observational skills, as calculated by the total score, improved significantly between the preintervention and postintervention tests (mean score 22.75 vs 33.5, respectively, *p* = 0.00005). This improvement in test score was also observed when focusing on art imagery (mean score 8 vs 12.5, respectively, *p* = 0.0003) and clinical imagery (mean score 14.75 vs 20.25, respectively, *p* = 0.0015) (Figure 4).

**Figure 5:**
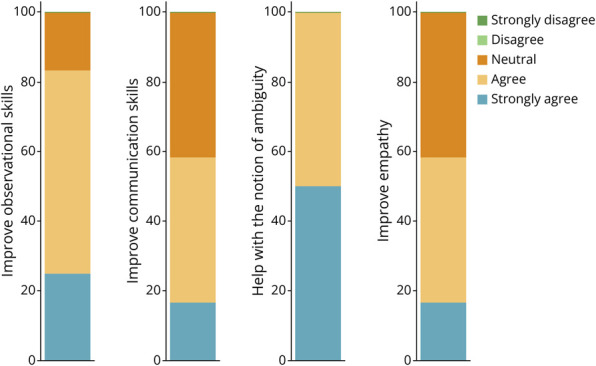
Graphs Representing 5-Point Likert Scale Values From Postintervention Surveys Residents were asked about the MANET project's effects on observational skills, communications skills, empathy, and their degree of comfort with the notion of ambiguity. MANET = Museum Art in Neurology Education Training.

Most residents noted a subjective improvement in their communication and observational skills and an increase in their empathy skills. One hundred percent of residents noted feeling more comfortable with the notion of ambiguity in a clinical setting after attending the museum sessions (Figure 5), and 100% of residents agreed that this project should be repeated for future years.

## Discussion

Residents' observational skills improved significantly throughout the academic year. These results are consistent with those documented in the existing literature across multiple medical specialties and levels of training. Most of the available literature regarding integrating visual arts in medical training relates to medical students.^9-12^ Similar programs were implanted in dermatology, ophthalmology, and radiology residencies, all specialties where visual diagnostic skills are key.^13-15^

Art-based curriculums have previously been developed in neurology residencies, using both narrative medicine and visual thinking exercise.^16^ Our study is unique in that it provided an objective tool to quantify and measure the improvement in observational skills.

One might argue that residents' scores improved with time because of their medical training. Our cohort included residents from multiple years of postgraduate training. When focusing specifically on each subsection, improvement in test scores was seen not only for residents' interpretations of clinical imagery but also art imagery. Test scores related to art imagery would not be expected to improve if underlying clinical training was considered the main driver in improvement in overall test scores. In addition, residents were also more likely to incorporate detailed and specific visual language learned during the museum art sessions (including terms such as symmetry, size, shape, and contrast) into their postintervention descriptions (eTable 3) for both clinical and art imagery. This suggests that the exposure to visual arts has a role in honing diagnostic and observational skills.

**Figure 6:**
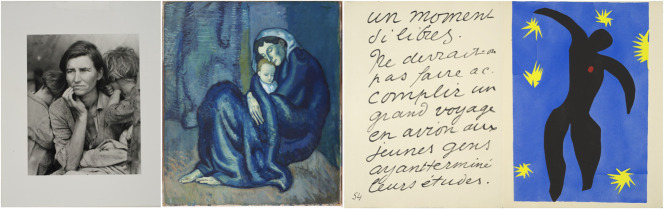
Examples of Artwork Presented During the Sessions at the Fogg Museum Left: Dorothea Lange, *Migrant Mother*, Nipomo, California, Harvard Art Museums/Fogg Museum, Transfer from the Carpenter Center for the Visual Arts, Photo © President and Fellows of Harvard College, 2.2002.705. Middle: Pablo Ruiz Picasso, *Mother and Child*, Harvard Art Museums/Fogg Museum, Bequest from the Collection of Maurice Wertheim, Class of 1906, © 2024 Estate of Pablo Picasso/Artists Rights Society, New York, Photo © President and Fellows of Harvard College, 1951.57. Reprinted with permission from the Harvard Art Museums. Right: Henri Matisse, *Icarus*, Harvard Art Museums/Fogg Museum, Francis H. Burr Memorial Fund, © 2024 Succession H. Matisse/Artists Rights Society, New York, Photo © President and Fellows of Harvard College, M12164. Reprinted with permission from the Harvard Art Museums.

Art and humanities can enrich and complement residency curriculum,^17^ but beyond any potential educational benefit, they offer a unique and safe space for self-reflection and personal growth. Art allows for the expression of emotions that may be difficult to convey with words alone.^17^ Because art has the potential to capture an expansive breadth of human experience, it may also help medical trainees better grapple with suffering, inequity, and other limitations of the health care system. During museum sessions, residents engaged with artworks like Picasso's *Mother and Child* painting and Dorothea Lange's *Migrant Mother* photograph (Figure 6). The 2 paintings use contrasting styles to offer intimate and humanizing portraits of both motherhood and poverty. During these sessions, the artworks elicited an emotional response from the participants and triggered conversations around both marginalized communities and the ways in which the experiences depicted in these artworks echoed the experiences of the residents' own patients at Boston Medical Center, a safety net hospital that cares primarily for medically underserved patients.

All residents noted feeling more comfortable with the notion of ambiguity in a clinical setting after the museum sessions. Henri Matisse's book *Jazz*, likely named after the improvisational and dynamic nature of the artwork, was chosen to explore the theme of ambiguity. This famous series of paper cutouts—characterized by vibrant, bold colors, abstract composition, and a sense of spontaneity and improvisation—mirrored the complexity and unpredictability of neurologic cases. To deepen residents' exploration of ambiguity, a hands-on session was also created, and residents were asked to improvise compositions with the same paper cutout method that Henri Matisse used to produce his book *Jazz*. Ambiguity is inherent to medical practice.^18^ Neurology residents are confronted every day with complex and challenging medical cases, and although it is natural for them to seek clear-cut answers, such answers may not be readily available in routine clinical practice. The ability to accept and manage ambiguity in health care is a crucial skill for physicians.^19^ Studies have shown that physicians with a low tolerance for ambiguity have higher levels of burnout^20^ and work stress^18^ and tend to over-refer and overutilize diagnostic tests.^21^ Acceptance of ambiguity in health care has also been found to have an effect on caring for end-of-life patients.^22^ Art, and humanities as a whole, can challenge this desire for cognitive closure and help physicians embrace ambiguity^23^ as an essential aspect of the human experience.

This study has several strengths. First, the museum art sessions were taught by professional art educators and facilitators. Each session was custom-designed and included a carefully curated selection of artwork intended to resonate with neurology residents. Another strength of this study was the use of a priori grading rubrics, which allowed for standardized grading. Answers were anonymous, and tests were linked by a personal, resident-chosen identifier. The program was very well-received by the residency program: 100% of residents who were included in the study agreed that it should be repeated for future years.

There are however several limitations to consider. Our sample study was relatively small, with only 12 residents included in this study. Although most residents attended at least 2 of the 3 art sessions, only 2 residents were able to attend all 3 sessions. Although every effort is made for all residents to attend didactics during their clinical rotations at the Boston Medical Center, residents typically miss didactic sessions during away rotations, vacations, and night float rotations. Tests were graded by a single author, and therefore, we were unable to calculate an interrater reliability. In addition, the assessment tool has not been formally validated yet. Future studies should prioritize the validation of this assessment tool to strengthen its scientific and practical applications. Another important limitation of the study was the absence of a control group. Although our findings suggest a benefit from our program, it is possible that engaging with another artistic medium (e.g., literature or music) would have a similar effect on residents' training. We believe that our study is a promising pilot for future studies investigating and comparing ways to enhance the humanistic aspects of residency training. Finally, our study focused specifically on evaluating observational skills preintervention and immediately postintervention and was not designed to determine whether the project had any lasting effect. Future research could benefit from including long-term follow-up. This approach could provide valuable insights not only into the durability of the effect but also into how residents are able to apply their observational skills in clinical practice.

Despite these limitations, this study emphasizes the importance of humanities in neurology training. Art can further develop residents' observational skills, foster their empathy and humanity, provide them with a safe space for self-reflection and personal growth, and offer them one of the most vital resources in medical training: time to slow down, observe, reflect, and analyze. Teaching artistic observation to neurology residents contributes to the development of well-rounded physicians with the capacity to be both skilled clinicians and compassionate healers.

## Appendix Authors

**Table:**

Name	Location	Contribution
Tatiana Greige, MD	Department of Neurology, Boston Medical Center, MA	Drafting/revision of the manuscript for content, including medical writing for content; major role in the acquisition of data; study concept or design; analysis or interpretation of data
David Odo, DPhil	Georgia Museum of Art, Athens	Drafting/revision of the manuscript for content, including medical writing for content
Camran Mani, MA	Harvard Art Museums, Cambridge, MA	Drafting/revision of the manuscript for content, including medical writing for content
Stephanie Bissonnette, DO, MPH	Department of Neurology, Virginia Commonwealth University, Richmond	Drafting/revision of the manuscript for content, including medical writing for content
Pria Anand, MD	Department of Neurology, Boston Medical Center, MA	Drafting/revision of the manuscript for content, including medical writing for content

## Glossary

MANET: Museum Art in Neurology Education Training
